# Macroalgal Peptides with Predicted α-Glucosidase Inhibitory Activity: Preparation and Molecular Docking

**DOI:** 10.3390/md24030091

**Published:** 2026-02-26

**Authors:** Sakhi Ghelichi, Seyed Hossein Helalat, Mona Hajfathalian, Birte Svensson, Charlotte Jacobsen

**Affiliations:** 1 Research Group for Bioactives–Analysis and Application, National Food Institute, Technical University of Denmark, 2800 Kongens Lyngby, Denmark; 2 Department of Health Technology, Technical University of Denmark, 2800 Kongens Lyngby, Denmark; 3 Enzyme and Protein Chemistry, Department of Biotechnology and Biomedicine, Technical University of Denmark, 2800 Kongens Lyngby, Denmark

**Keywords:** *Palmaria palmata*, α-glucosidase inhibition, Alcalase^®^, low-molecular-weight peptides, in silico analysis, molecular docking

## Abstract

This study investigated the α-glucosidase inhibitory potential of enzymatic/alkaline treatments from *Palmaria palmata* using different proteases and pairwise combinations thereof. Treatments prepared with Alcalase^®^, Flavourzyme^®^, and Formea^®^ Prime, alone or in combination, were evaluated for dose-dependent inhibitory activity. Alcalase^®^-derived treatments exhibited the highest α-glucosidase inhibition, achieving an IC_50_ of 2.48 mg·mL^−1^, outperforming other treatments and combinations. Membrane fractionation of the Alcalase^®^-derived treatment into >5 kDa, 3–5 kDa, 1–3 kDa, and <1 kDa fractions revealed a size-dependent trend, with the <1 kDa fraction showing the strongest inhibition (IC_50_ of 1.94 mg·mL^−1^). Three peptides, RADIPFRRA, DGIAEAWLG, and FWSQIFGVAF, from the <1 kDa fraction were identified as potential α-glucosidase inhibitors using the BIOPEP-UWM database and were further selected based on a Peptide Ranker score above 0.6 for in silico docking analyses. Docking revealed distinct binding modes: RADIPFRRA and DGIAEAWLG occupied the catalytic cleft, interacting with key residues (Asp518, Asp616, Trp481, Trp613) consistent with competitive inhibition, whereas FWSQIFGVAF bound to a peripheral site, suggesting potential allosteric modulation. Physicochemical analysis further highlighted differences in charge and isoelectric point correlating with their binding behavior. Together, these findings demonstrate that low-molecular-weight peptides derived from *P. palmata* proteins, particularly those generated by Alcalase^®^, possess significant α-glucosidase inhibitory activity, and provide structural insights for the rational design of peptide-based modulators of carbohydrate metabolism.

## 1. Introduction

Diabetes mellitus is a major global metabolic disorder characterized by chronic hyperglycemia and associated with progressive complications affecting multiple organ systems, highlighting the critical importance of effective glycemic control in disease management [1]. Type 2 diabetes, which accounts for more than 90% of diabetes cases globally, is a multifactorial condition marked by insulin resistance and reduced pancreatic β-cell function, leading to impaired glucose uptake and sustained hyperglycemia, a major driver of the disease’s secondary complications [2]. Treatment of type 2 diabetes typically combines lifestyle changes with medication. Although several drugs are available to manage elevated blood glucose, many are expensive and may cause significant adverse effects [3]. For instance, glucagon-like peptide 1 (GLP-1) receptor agonists have been linked to an increased risk of kidney damage [4]. These drawbacks have encouraged the search for safer, diet-based alternatives derived from natural sources [5].

Enzymes involved in the regulation of post-meal glucose spikes have been identified as important therapeutic targets [6]. Among these targets, α-glucosidase acts at the intestinal brush border as the final enzyme in carbohydrate digestion, catalyzing the release of absorbable glucose, making its inhibition an effective strategy for limiting postprandial elevations in blood glucose levels [7]. Conventional α-glucosidase inhibitors (e.g., acarbose, voglibose, and miglitol) work by slowing carbohydrate digestion, but their use is often constrained by unpleasant gastrointestinal side effects [4]. As a result, there is strong motivation to discover natural, safer alternatives that can inhibit α-glucosidase with fewer complications [8]. In recent years, bioactive peptides have attracted growing interest as α-glucosidase inhibitors, particularly due to their high target specificity, strong binding capacity, and generally low toxicity, making them appeal to both food and pharmaceutical research communities [9]. Food-derived peptides, which are encrypted within parent protein sequences and released during digestion or processing, have been shown to interact with enzyme active sites thus exerting diverse biological functions, including modulation of glucose metabolism and improvement of glycemic control [10]. Therefore, there is a clear need to explore sustainable and underutilized natural protein sources as reservoirs of bioactive peptides with potential antidiabetic activity.

Marine macroalgae are increasingly recognized as sustainable sources of bioactive compounds with potential benefits in metabolic disorder management [11]. Red seaweed is characterized by relatively high protein content, making it a promising candidate for the generation of health-promoting peptides [12]. *Palmaria palmata*, a red macroalga approved for human consumption in the European Union, has attracted considerable interest due to its nutritional value and suitability for functional food applications [13]. Protein-rich treatments from *P. palmata* have been shown to exhibit diverse functional and bioactive properties [14,15]. Even the residues remaining after the treatment have been reported to retain functional and bioactive potential [16]. The treatments obtained from this species thus demonstrated anti-obesity and antidiabetic potential through the inhibition of metabolic enzymes [17,18]. Furthermore, purified peptides derived from *P. palmata* protein hydrolysates have previously been reported to modulate key digestive enzymes, including pancreatic lipase and pancreatic α-amylase [19], highlighting their potential anti-obesity and antidiabetic effects. Despite these promising findings, the α-glucosidase inhibitory potential of peptides from *P. palmata* remains largely unexplored, representing a critical knowledge gap in understanding their full metabolic regulatory capacity. Given the central role of α-glucosidase in carbohydrate digestion and postprandial glucose regulation, the present study aimed to evaluate α-glucosidase inhibitory activity of purified and identified peptides from *P. palmata* enzymatic/alkaline treatments. In addition, molecular docking analyses were employed to elucidate peptide-enzyme interactions, characterize binding modes, and provide mechanistic insight into how these peptides may modulate enzyme activity. This strategy enables a targeted assessment of bioactive sequences, laying the foundation for the rational design of functional food ingredients or peptide-based therapeutics aimed at glycemic control.

## 2. Results

### 2.1. α-Glucosidase Inhibitory Properties of Crude Treatments

The α-glucosidase inhibitory activity of enzymatic/alkaline treatments prepared from *P. palmata* using different proteases, combinations thereof, and a control (without any protease added) is presented in Table 1. Inhibition was measured at four concentrations (4, 2, 1, and 0.5 mg·mL^−1^), and IC_50_ values were calculated when 50% inhibition was achieved. Overall, the results indicate a clear dose-dependent inhibitory effect, with higher concentrations resulting in stronger enzyme inhibition. Within each treatment, significant differences in α-glucosidase inhibition were observed across concentrations (*p* < 0.05). The treatment using Alcalase^®^ alone resulted in the highest α-glucosidase inhibitory activity. At 4 mg·mL^−1^, inhibition reached 68.19%, which was significantly higher than all other single or protease combination treatments (*p* < 0.05). Its IC_50_ value of 2.48 mg·mL^−1^ further confirms its superior potency, as it was the only treatment achieving 50% inhibition at a concentration threshold of below 3 mg·mL^−1^. Flavourzyme^®^ and Formea^®^ Prime, when used individually, produced moderate inhibition of 41.24% and 33.30%, respectively, at 4 mg·mL^−1^, neither reaching an IC_50_ under the tested concentrations.

Two combination treatments of Alcalase^®^ + Flavourzyme^®^ and Alcalase^®^ + Formea^®^ Prime exhibited intermediate, 54.57% and 52.97% inhibition at 4 mg·mL^−1^, and corresponding IC_50_ values of 3.36 mg·mL^−1^ and 3.46 mg·mL^−1^, respectively. While these combinations enhanced α-glucosidase inhibitory activity relative to Flavourzyme^®^ or Formea^®^ Prime alone, they were less effective than Alcalase^®^ alone at the highest concentration (*p* < 0.05). Furthermore, except at 2 mg·mL^−1^ (*p* > 0.05), these combinations showed significantly lower α-glucosidase inhibition compared to Alcalase^®^ alone (*p* < 0.05). The lowest α-glucosidase inhibitory activities were observed in treatments that did not include Alcalase^®^. Among the non-Alcalase^®^ proteases, Flavourzyme^®^ exhibited higher α-glucosidase inhibition than Formea^®^ Prime, showing significantly greater activity than both Formea^®^ Prime and the control (no protease) at all tested concentrations (*p* < 0.05). These results strongly support the selection of Alcalase^®^-derived treatments for further fractionation and bioactivity-guided peptide characterization.

### 2.2. Ability of Different Molecular Weight Fractions of Alcalase^®^-Assisted Treatment to Inhibit α-Glucosidase

Among the parent enzymatic/alkaline treatments tested, three reached measurable IC_50_ values, with the most potent treatment exhibiting an IC_50_ of 2.48 mg·mL^−1^. This treatment, derived by using Alcalase^®^, was further fractionated using membrane fractionation into four molecular weight fractions: >5 kDa, 3–5 kDa, 1–3 kDa, and <1 kDa. The α-glucosidase inhibitory activity of these fractions at 4, 2, 1, and 0.5 mg·mL^−1^ is presented in Table 2, along with calculated IC_50_ value for the sample that reached 50% inhibition within the tested concentration range. A very clear size-dependent inhibitory trend was observed. It should be noted, however, that while molecular size appears to influence activity, other factors such as amino acid composition, sequence motifs, and hydrophobicity may also contribute to the observed differences among fractions. The <1 kDa fraction exhibited the highest α-glucosidase inhibition across all concentrations, reaching 70.10% at 4 mg·mL^−1^, with significant differences in inhibition across concentrations (*p* < 0.05). This fraction was the only one reaching IC_50_ within the tested concentration range, with a value of 1.94 ± 0.14 mg·mL^−1^. Statistical analysis using Welch’s unpaired two-tailed *t*-test indicated that the IC_50_ of the <1 kDa fraction was significantly lower than that of its parent treatment (t = 5.32, df = 3.70, *p* = 0.0075), demonstrating that fractionation enriched the active components.

The 1–3 kDa fraction showed moderate inhibition of 43.56% at 4 mg·mL^−1^ and significant differences across concentrations (*p* < 0.05) but did not reach IC_50_. The 3–5 kDa and >5 kDa fractions exhibited the lowest inhibitory activity, with maximal inhibition of 26.83% and 24.16%, respectively (*p* < 0.05 compared to <1 kDa and 1–3 kDa fractions), also showing significant differences across concentrations (*p* < 0.05), and neither reached IC_50_ within the tested range. Based on these findings, the <1 kDa fraction was selected for further purification, peptide identification, in silico analysis, and molecular docking of selected peptides.

### 2.3. In Silico Prediction of Bioactivity, Toxicity, and Allergenicity of Identified Peptides

All peptides identified from the <1 kDa fraction were analyzed for their potential biological activity using PeptideRanker. Out of 536 sequences evaluated, 51 peptides scored above 0.6, indicating a high likelihood of bioactivity (Supplementary Material Table S1). These 51 peptides were subsequently assessed for safety. ToxinPred analysis predicted them to be non-toxic. Potential allergenicity was evaluated using AllerCatPro 2.0 and AllergyPred. Based on these safety assessments, the selected peptides were considered suitable for further downstream analysis.

### 2.4. Molecular Docking of Selected α-Glucosidase-Inhibiting Peptides

The successfully validated peptides were screened for potential lysosomal acid α-glucosidase (GAA) inhibitory activity using the BIOPEP-UWM database. Afterwards, peptides with a Peptide Ranker score above 0.6, namely RADIPFRRA, DGIAEAWLG, and FWSQIFGVAF, were chosen for further docking and physicochemical analyses. Docking analyses were performed on these peptides to evaluate their binding modes and potential mechanisms of inhibition against GAA. All three peptides were found to bind to GAA, but their interaction profiles differed markedly.

**Figure 1:**
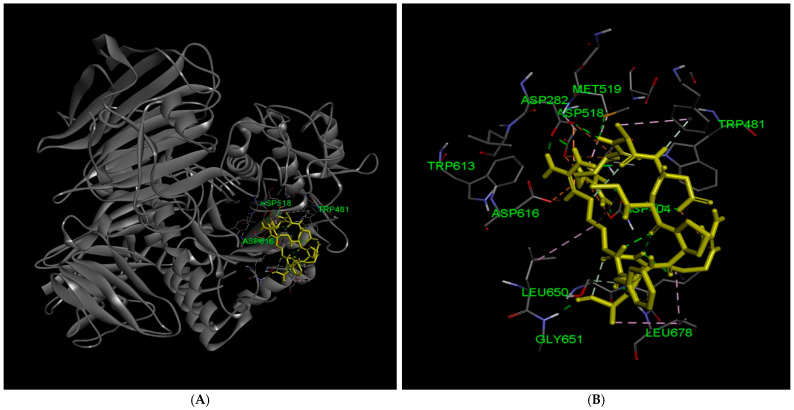

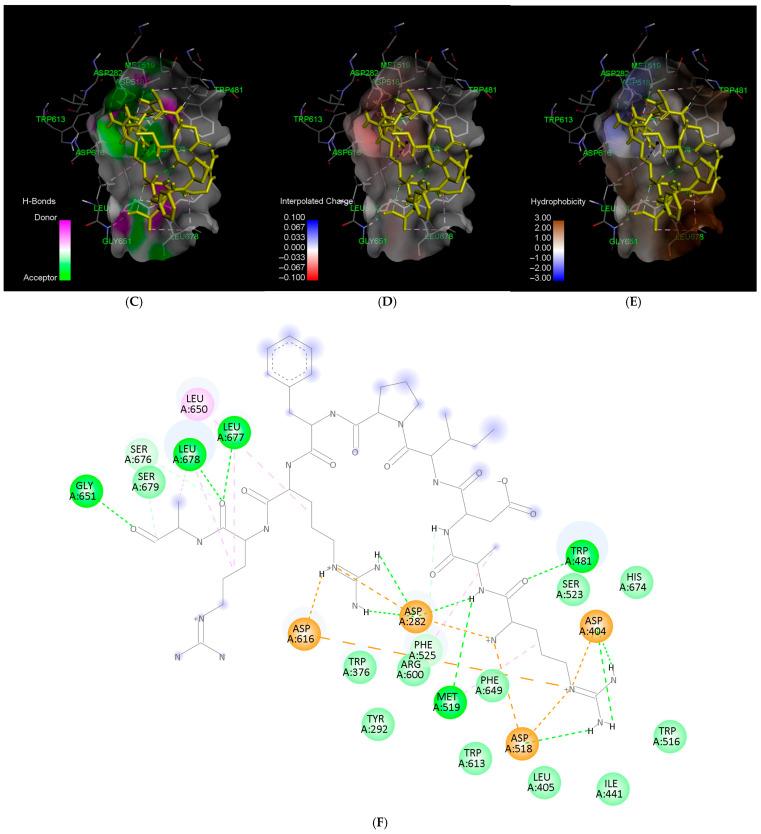
Docking analysis of the RADIPFRRA peptide with lysosomal acid α-glucosidase (GAA). (**A**) Overall docking pose of the RADIPFRRA peptide bound to GAA, showing peptide localization within the catalytic region of the enzyme; (**B**) Enlarged view of the pocket, illustrating the positioning of the peptide inside the catalytic cleft of GAA; (**C**) Distribution of hydrogen bond interactions between RADIPFRRA and residues of the enzyme, highlighting direct contacts within the active site pocket; (**D**) Electrostatic surface representation of the active site pocket, showing charge complementarity between the enzyme and the bound peptide; and (**E**) Hydrophobicity map of the active site pocket region, illustrating hydrophobic interactions that stabilize RADIPFRRA binding within the active site; and (**F**) Two-dimensional enzyme–peptide interactions. Interacting residues are color-coded based on interaction type: light green indicates van der Waals interactions, dark green dashed lines represent conventional hydrogen bonds, pale green dashed lines indicate carbon hydrogen bonds, orange dashed lines represent salt bridge interactions, yellow dashed lines indicate attractive charge interactions, pink dashed lines represent alkyl or π-alkyl interactions, and purple circles indicate atoms directly involved in hydrogen bond or polar interactions in the docking model.

RADIPFRRA binds directly within the catalytic cleft, interacting with essential catalytic residues Asp518 and Asp616, as well as substrate-recognition residues Trp481 and Trp613 [20]. Additional contacts with residues lining the pocket, including Met519, Ile441, Leu650, Leu677, Leu678, Ser676, and Gly651, indicate that the peptide occupies both the core catalytic site and its entrance, consistent with competitive inhibition. The binding pattern suggests that RADIPFRRA both physically blocks substrate access and stabilizes the local conformation of the catalytic pocket, potentially restricting the flexibility of adjacent loop regions facilitating carbohydrate hydrolysis. Hydrogen bonding and van der Waals interactions with polar and non-polar residues along the pocket further anchor the peptide, enhancing residence time within the active site. These multiple points of contact imply that RADIPFRRA may interfere with substrate positioning and catalysis, highlighting an inhibitory mechanism combining steric obstruction of substrate access and direct interaction with the catalytic machinery (Figure 1).

**Figure 2:**
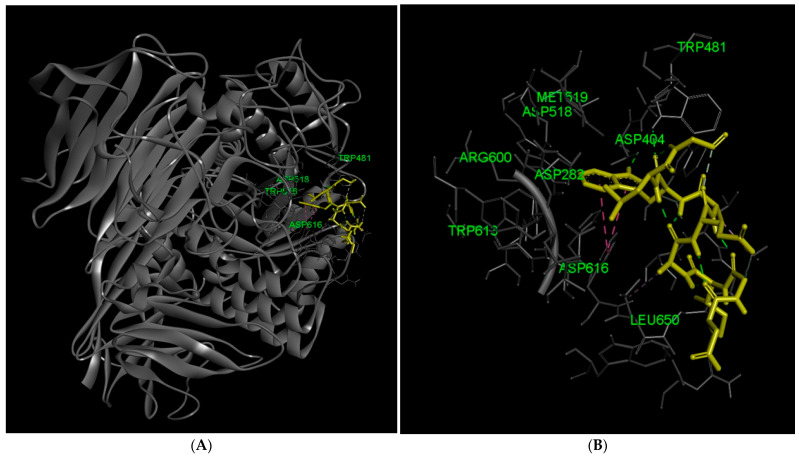

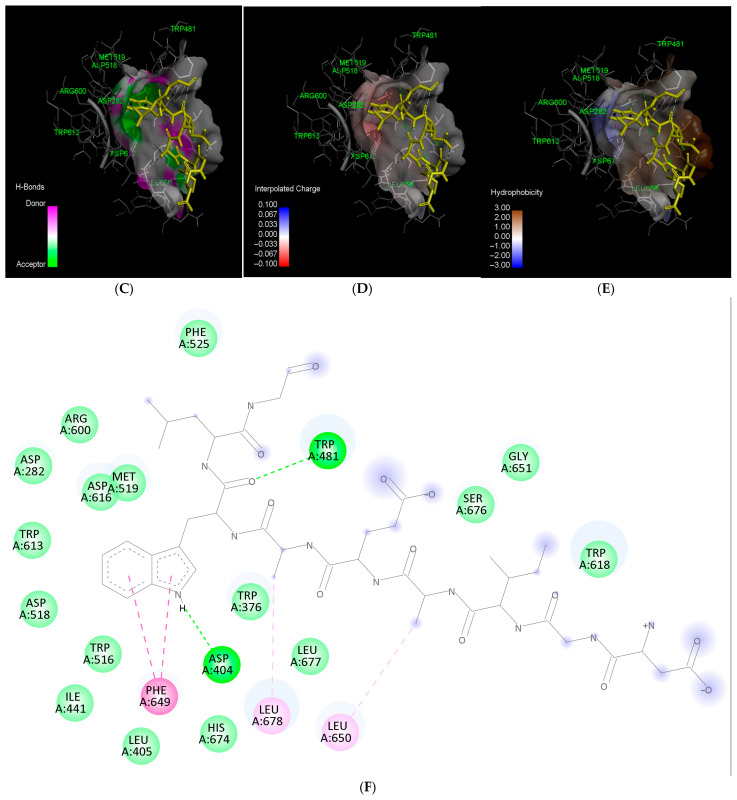
Docking analysis of the DGIAEAWLG peptide with lysosomal acid α-glucosidase. (**A**) Overall docking pose of the DGIAEAWLG peptide bound to GAA, showing peptide localization within the catalytic region of the enzyme; (**B**) Enlarged view of the pocket, illustrating the positioning of the peptide inside the catalytic cleft of α-glucosidase; (**C**) Distribution of hydrogen bond interactions between DGIAEAWLG and residues of the enzyme, highlighting direct contacts within the active site pocket; (**D**) Electrostatic surface representation of the active site pocket, showing charge complementarity between the enzyme and the bound peptide; and (**E**) Hydrophobicity map of the active site pocket region, illustrating hydrophobic interactions that stabilize DGIAEAWLG binding within the active site; and (**F**) Two-dimensional enzyme-peptide interactions. Residues interacting with the peptide are colored according to the type of interaction. Light green indicates van der Waals interactions, green dashed lines represent conventional hydrogen bonds, pink dashed lines indicate π-π stacked interactions, light pink dashed lines represent alkyl or π-alkyl interactions, and purple circles indicate atoms directly involved in hydrogen bond or polar interactions in the docking model.

DGIAEAWLG also targets the catalytic cleft, interacting with Asp518, Asp616, Trp481, Trp516, Trp613, and Arg600. The peptide further engages multiple residues, including Met519, Ile441, Phe525, Phe649, Leu650, Leu677, Leu678, Asp404, Asp282, and His674, suggesting deep insertion and extensive stabilization within the active site. The involvement of multiple aromatic residues within the pocket indicates close packing of the peptide against the catalytic framework, which may restrict access of substrate to the active site. In addition, contacts with both catalytic and peripheral pocket residues suggest that DGIAEAWLG spans a substantial portion of the binding cleft, forming a dense interaction network that anchors the peptide within the enzyme. Together, these features are consistent with a competitive inhibition mechanism and indicate potentially stronger inhibitory behavior relative to RADIPFRRA, based on the extent and distribution of active-site interactions (Figure 2).

**Figure 3:**
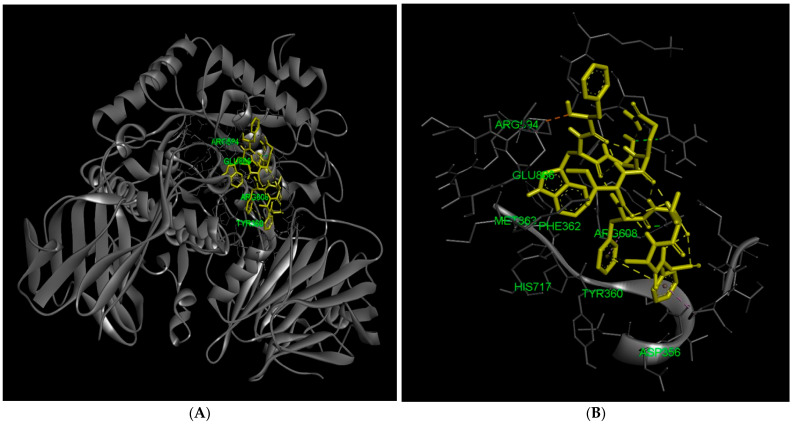

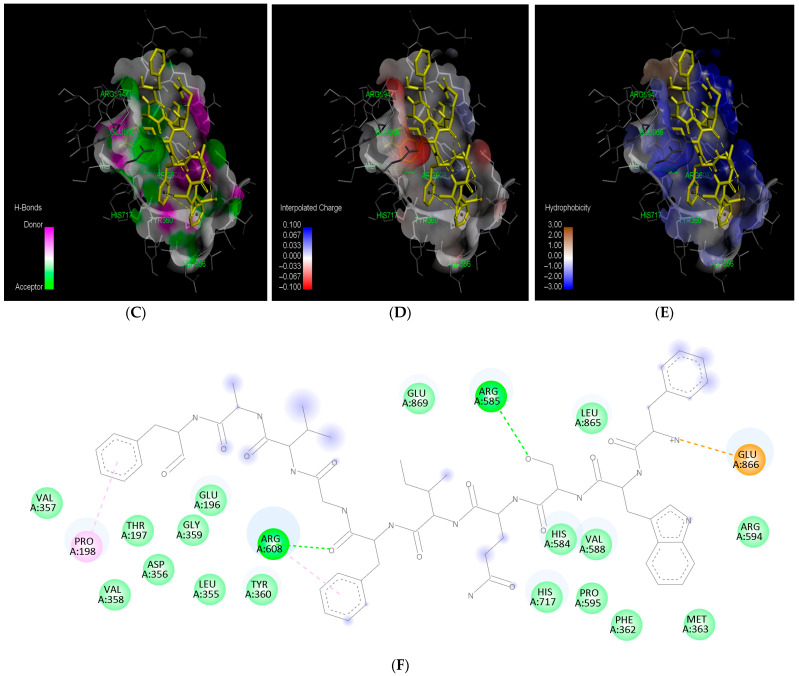
Docking analysis of the FWSQIFGVAF peptide with lysosomal acid α-glucosidase. (**A**) Overall docking pose of the FWSQIFGVAF peptide bound to GAA; (**B**) Enlarged view of the binding site, illustrating the positioning of the peptide; (**C**) Distribution of hydrogen bond interactions between FWSQIFGVAF and the residues of the enzyme, highlighting direct contacts within at the surface binding site; (**D**) Electrostatic surface representation, showing charge complementarity between the enzyme and the bound peptide; and (**E**) Hydrophobicity map of the surface binding site, illustrating hydrophobic interactions that stabilize FWSQIFGVAF binding within the binding site; and (**F**) Two-dimensional enzyme-peptide interactions. Interacting residues are color-coded based on interaction type: light green indicates van der Waals interactions, dark green dashed lines represent conventional hydrogen bonds, orange dashed lines indicate attractive charge interactions, pink dashed lines represent alkyl or π-alkyl interactions, and purple circles indicate atoms directly involved in hydrogen bond or polar interactions in the docking model.

In contrast, FWSQIFGVAF binds to a peripheral region of GAA, interacting with surface-exposed charged residues such as Arg594, Arg585, Arg608, Glu866, Glu869, and Glu196, along with hydrophobic contacts involving Tyr360, Phe362, Met363, Leu355, Val357, Val358, Val588, and Leu865. The localization of these interactions away from the catalytic cleft indicates that the peptide does not directly obstruct substrate binding or interact with catalytic residues. Instead, the interaction network is confined to solvent-accessible areas of the enzyme, suggesting that FWSQIFGVAF associates with GAA through surface recognition rather than active-site insertion. Binding at a surface position may influence enzyme function indirectly by modulating local conformational dynamics or restricting structural flexibility required for efficient catalysis. Collectively, this interaction pattern supports a potential allosteric or non-competitive inhibitory mechanism, distinct from the competitive binding observed for peptides occupying the catalytic pocket (Figure 3).

### 2.5. Physicochemical Characterization of Selected α-Glucosidase-Inhibiting Peptides

The physicochemical properties: molecular weight, isoelectric point (pI), and net charge at physiological pH differ for the selected peptides RADIPFRRA, DGIAEAWLG, and FWSQIFGVAF (Table 3), providing sequence-based insights into their behavior in aqueous environments and their potential interactions with α-glucosidase. RADIPFRRA exhibited a high pI (11.70) and a net positive charge (+1.76) at pH 7, whereas DGIAEAWLG showed a low pI (3.67) and a net negative charge (−2.24). Furthermore, FWSQIFGVAF displayed a moderately low pI (5.52) and was electrically near-neutral (−0.24) at physiological pH. The peptides slightly varied in length and aromatic residue content, which may influence their conformational flexibility and interaction patterns with protein targets. Overall, these physicochemical differences highlight the structural diversity among the selected peptides and provide a basis for interpreting their distinct binding modes observed in docking analyses.

## 3. Discussion

The present study demonstrated that *P. palmata* treatments produced through Alcalase^®^ hydrolysis followed by alkaline extraction exhibit significantly greater α-glucosidase inhibitory activity than treatments generated with Flavourzyme^®^ and Formea^®^ Prime alone, combined or their individual combination with Alcalase^®^, or the control without protease (*p* < 0.05). This result highlights *P. palmata* as a promising source of proteins and peptides that can be selectively released through enzymatic processing to generate compounds having antidiabetic potential [21]. Among all protease/alkaline treatments, the Alcalase^®^-derived treatment gave the lowest IC_50_, indicating its stronger capacity to inhibit α-glucosidase. These findings emphasize the critical impact of protease selection on the generation of bioactive compounds with potential antidiabetic properties [22,23]. The enhanced inhibitory activity of the Alcalase^®^ treatment aligns with previous reports on Alcalase^®^-generated peptides exhibiting potent inhibitory effects against carbohydrate-digesting enzymes acting on algal and other plant-based protein sources [24,25,26,27]. This trend may be associated with the broad endoprotease specificity of Alcalase^®^, which promotes extensive protein cleavage and has been reported to increase essential amino acid availability in treatments [28]. As a serine protease, Alcalase^®^ preferentially cleaves internal peptide bonds adjacent to hydrophobic residues, leading to the release of shorter, hydrophobic peptides [29]. Hydrophobic and aromatic amino acids have been implicated in stronger interactions with enzyme active sites through hydrophobic contacts and hydrogen bonds, which can improve inhibitory efficiency [4,30]. Alcalase^®^ has also been reported to produce a higher degree of protein hydrolysis compared with Flavourzyme^®^ [31], resulting in a greater release of low-molecular-weight peptides, which may further contribute to enhanced α-glucosidase inhibition [1,32]. In contrast, Flavourzyme^®^ that contains both endo- and exopeptidase activities produces a wider distribution of peptide sizes [33], potentially dispersing sequences with inhibitory potential across a broader molecular size range. Therefore, the peptides obtained by Flavourzyme^®^ mediated treatment may be less rich in structural motifs relevant for α-glucosidase binding accounting for its moderate inhibitory profile. Similarly, treatments using Formea^®^ Prime had the lowest inhibitory activity among the enzymatic treatments. This outcome is consistent with our prior observations that Formea^®^ Prime produced fewer α-glucosidase inhibitory sequences in comparable seaweed matrices [19], possibly due to a more restricted substrate specificity and reduced capacity to liberate peptide sequences critical for enzyme interaction. The moderately enhanced inhibitory activity found for combination treatments (Alcalase^®^ + Flavourzyme^®^ and Alcalase^®^ + Formea^®^ Prime) relative to single treatments using Flavourzyme^®^ or Formea^®^ Prime alone highlights the dominant contribution of Alcalase^®^ treatments to α-glucosidase inhibition rather than a strong protease synergistic effect. While the inclusion of Alcalase^®^ improved inhibitory activity compared with non-Alcalase^®^ treatments, the combinations did not surpass the inhibition achieved by Alcalase^®^ alone, suggesting that additional proteases provided limited complementary effects. This pattern may reflect overlapping or non-complementary cleavage specificities during the combined protease hydrolysis, resulting in a peptide ensemble in which the relative abundance of highly active sequences generated by Alcalase^®^ is reduced compared with Alcalase^®^-only treatments. This trend, whereby Alcalase^®^-treated samples exhibited superior bioactive properties, emphasizes the effectiveness of Alcalase^®^ in generating and releasing peptides with enhanced bioactivity from algal protein matrix [21,34,35]. Importantly, the dose-dependent inhibitory profiles across all treatments underline the concentration sensitivity of α-glucosidase inhibition by seaweed-derived peptides [36]. The steep increases in inhibition with rising peptide concentration suggest a high density of inhibitory sequences in the Alcalase^®^ treatment, supporting its utility in functional food or nutraceutical formulations designed to moderate postprandial hyperglycemia. Taken together, these findings support the selection of Alcalase^®^ hydrolysates as a promising source of α-glucosidase inhibitory peptides. The superior inhibitory performance of Alcalase^®^, relative to Flavourzyme^®^, Formea^®^ Prime, treatments and their combinations, illustrates the importance of protease screening and choice in bioactive peptide generation from complex protein substrates [37]. This sets a strong foundation for targeted peptide isolation and characterization in subsequent analytical stages.

The α-glucosidase inhibitory activity of the Alcalase^®^-derived fractions exhibited a clear molecular weight-dependent pattern. Among the four molecular weight fractions, the <1 kDa fraction demonstrated the strongest activity, reaching 70.10% inhibition at 4 mg·mL^−1^ and the only fraction to achieve a measurable IC_50_ (1.94 mg·mL^−1^). The 1–3 kDa fraction showed moderate inhibition, while the 3–5 kDa and >5 kDa fractions displayed limited inhibitory activity. This observed peptide molecular weight-dependent inhibition of α-glucosidase clearly demonstrates the purposeful efficacy of low molecular weight peptides in algal treatments prepared using enzymatic/alkaline processing [37,38]. Small peptides generally exhibit enhanced accessibility to the active site of targeted enzymes, increased conformational flexibility, and favorable physicochemical properties, for example good solubility even with high content of hydrophobic residues, which collectively facilitate stronger interactions with catalytic and allosteric regions [1,39]. These properties likely underpin the superior inhibitory potency of the <1 kDa fraction and are consistent with previous reports demonstrating that short-chain peptides from various protein sources, including algal proteins, are more effective at modulating carbohydrate-digesting enzymes [19,40,41]. Intermediate-sized peptides (1–3 kDa) retained moderate activity, suggesting that certain sequences within this range still contribute to inhibition, whereas larger peptides (3–5 kDa and >5 kDa) were comparatively less inhibitory. This pattern reinforces the notion that molecular size alone is a critical determinant of bioactivity, although other structural features including amino acid composition, sequence motifs, charge distribution, and hydrophobicity also influence enzyme interactions [42]. The enrichment of inhibitory activity following the fractionation demonstrates the effectiveness of Alcalase^®^ in generating bioactive peptides from the parent red macroalgal protein matrix. These findings align very well with previous studies indicating that targeted enzymatic hydrolysis can selectively produce peptide fractions with enhanced functional properties, consolidating the coupling of protease selection with molecular weight-based fractionation for the development of functional ingredients [43]. From a broader perspective, the present identification of low molecular weight peptides with potent α-glucosidase inhibitory activity accentuates the potential of *P. palmata*-derived treatments as natural modulators of carbohydrate metabolism [19,44,45]. Such bioactive peptides may contribute to managing postprandial hyperglycemia, complementing dietary strategies for metabolic health. It should be noted that the present study focused primarily on inhibitory potency rather than quantitative peptide distribution among fractions. It remains possible that highly active peptides are present in relatively minor proportions, and that the hydrolysis conditions employed may not fully maximize their release. Enzymatic specificity, degree of hydrolysis, and processing parameters are known to influence peptide profiles. Therefore, further optimization of hydrolysis conditions could potentially increase the recovery of peptides with enhanced lipase inhibitory activity.

The peptides RADIPFRRA and DGIAEAWLG were traced back to the photosystem II CP47 reaction center protein, whereas FWSQIFGVAF originated from the photosystem II D2 protein. Both parent proteins are integral components of the photosystem II (PSII) core complex, where they play essential roles in light harvesting, electron transfer, and stabilization of the photochemical reaction center in red algae. These PSII-associated proteins are characterized by structurally constrained domains and a high degree of evolutionary conservation, reflecting their critical roles in energy conversion processes. Enzymatic hydrolysis of such membrane-associated and functionally dense proteins may therefore release peptide fragments with distinct structural features and interaction capabilities. The identification of α-glucosidase inhibitory peptides derived from PSII core proteins suggests that photosynthetic protein complexes represent a previously underexplored source of bioactive sequences with potential metabolic regulatory functions [46]. The docking and physicochemical analyses collectively reveal that peptide sequence and structural properties critically shape how inhibitors interact with lysosomal acid α-glucosidase. The binding of inhibitory peptides to α-glucosidase at distinct sites is predicted to be consistent with potential α-glucosidase inhibition interaction, reported in previous studies, where various modes of inhibition have been observed to modulate of enzyme activity [47]. Previous studies suggest that peptides predicted to engage the catalytic cleft may partially obstruct substrate binding, forming stable interactions with catalytic residues and adjacent substrate recognition sites [1]. Peptides such as RADIPFRRA and DGIAEAWLG that interact with the two catalytic Asp residues and aromatic substrate recognizing residues in GAA are predicted to potentially interfere with substrate binding based on docking. Food-derived α-glucosidase inhibitory peptides frequently bind in or near the active site, forming hydrogen bonds and hydrophobic contacts, for instance with residues analogous to Asp518 and Asp616 in α-glucosidase, which may reduce substrate accessibility according to docking predictions [1,48,49]. These interaction patterns align with classical competitive inhibition kinetics, where inhibitor binding to the active site pocket directly interferes with catalysis and increases apparent *K*_m_ (substrate concentration at half-maximal velocity) without affecting *V*_max_ (maximum reaction velocity) [50], though experimental confirmation is needed. The physicochemical characteristics correlated with active-site binding also reflect broader sequence-activity relationships observed in the literature. Charged side chains such as arginine and lysine can form electrostatic interactions with negatively charged pockets in α-glucosidase, stabilizing peptide-enzyme complexes [41]. Hydrophobic and aromatic side chains enhance van der Waals and stacking interactions with aromatic residues within the catalytic cleft, as seen in diverse peptide–α-glucosidase systems [1]. These properties support stronger binding affinities and help explain why peptides predicted to engage active-site hot spots tend to exhibit higher docking scores and, in some cases, lower experimentally observed IC_50_ values. By contrast, FWSQIFGVAF exemplifies a non-active-site binding mode, where interactions are predominantly with peripheral or surface residues. Such binding is predicted to resemble peripheral or allosteric-like interactions suggested in other peptide-enzyme systems, where ligands bind outside the catalytic pocket but still alter enzyme function through conformational modulation or hinder substrate turnover indirectly [41]. Allosteric inhibition can be particularly relevant when substrates are abundant, as peripheral binding may modulate enzyme conformational dynamics or accessibilities without directly competing with substrate molecules, a property that is mechanistically distinct from classical competitive inhibition [51,52].

The biological implications of distinct inhibitory mechanisms are important for designing functional peptide modulators. Competitive inhibitors are typically more effective at low substrate concentrations, where they directly obstruct substrate access to the active site [53]. Allosteric or peripheral modulators may offer advantages under physiological conditions where substrate concentrations vary, as they can fine-tune enzyme activity without necessarily competing directly with substrate binding [41]. This mechanistic diversity suggests that a rational design strategy for peptide-based α-glucosidase modulators can leverage both active-site and allosteric binding modes to achieve context-dependent modulation of enzymatic activity. Furthermore, while molecular docking provides valuable predictive insights into binding orientations and interaction energies, it should be interpreted in conjunction with experimental kinetics and structural studies [19]. Docking predictions have been successfully correlated with experimental inhibitory activities in numerous α-glucosidase studies, indicating that lower binding energy and involvement of key catalytic residues often translate into stronger in vitro inhibition [54,55,56,57,58]. However, in the present study, no experimental kinetic analyses were performed, and docking alone cannot fully capture dynamic conformational changes or solvent effects that may influence actual inhibitory behavior. In addition, the α-glucosidase inhibitory activity of the most promising peptides identified here has not yet been confirmed using chemically synthesized peptides. Therefore, complementary studies such as kinetic assays and experimental testing of synthetic peptides are necessary to validate both the predicted binding interactions and the inhibitory potency of the peptides under physiological conditions [59]. It should be noted that the in vitro inhibition assays were conducted using α-glucosidase from *Saccharomyces cerevisiae*, a widely used screening model, whereas molecular docking was performed using human lysosomal acid α-glucosidase (GAA). These enzymes differ substantially in sequence, structure, and physiological role. As such, the docking results provide only preliminary insights into potential peptide-enzyme interactions and should not be interpreted as directly predictive of postprandial glucose regulation in humans. Nonetheless, the docking analysis highlights residues and structural regions that may contribute to inhibitory activity and can guide future studies using human intestinal α-glucosidases. Overall, the diverse inhibitory binding modes observed in this study reinforce the idea that peptide-enzyme interactions are governed by a combination of sequence-specific contacts, structural complementarity, and physicochemical compatibility. Incorporating both active-site and peripheral peptides into future design frameworks may expand the repertoire of functional modulators of carbohydrate-processing enzymes, with potential applications in dietary management of glycemic response or therapeutic development.

## 4. Materials and Methods

### 4.1. Enzymes and Reagents

The commercial proteases Alcalase^®^ (2.4 amino acid units [AU-A] g^−1^), Flavourzyme^®^ (500–1000 leucine aminopeptidase units [LAPU] g^−1^), and Formea^®^ Prime (140 kilo mannitol units [KMTU] g^−1^) were supplied by Novenesis A/S (formerly Novozymes A/S, Bagsværd, Denmark). All organic solvents used for chromatographic analyses were of HPLC grade and sourced from Lab-Scan (Dublin, Ireland). Ultrapure water was produced in-house at DTU Food using a Milli-Q^®^ Advantage A10 purification system (Millipore Corporation, Billerica, MA, USA). The α-glucosidase from *S. cerevisiae* was purchased from Sigma-Aldrich (Steinheim, Germany). All remaining reagents and chemicals were obtained from Merck (Darmstadt, Germany).

### 4.2. Macroalgal Biomass Preparation

Dried macroalgal biomass of *P. palmata*, harvested along the Faroe Islands coastline between late autumn and early winter 2023, was procured from a commercial supplier (Dansk Tang, Nykøbing Sj., Denmark). Prior to treatment, the material was subjected to freeze-drying using a ScanVac CoolSafe system (LaboGene A/S, Allerød, Denmark) to ensure moisture removal. The dried seaweed was subsequently milled using a laboratory-scale grinder (KN 295 Knifetec^TM^, Foss A/S, Hillerød, Denmark) to approximately 0.5–1.0 cm particle size, and stored in sealed plastic bags at −20 °C and protected from light until further use.

### 4.3. Preparation of Crude Macroalgal Treatments

Crude macroalgal treatments were generated using a combined enzymatic hydrolysis and alkaline extraction strategy. Briefly, seaweed powder (5 g) was dispersed in deionized water (100 mL; solid-to-liquid ratio 1:20, *w*/*v*) in blue-capped bottles, prepared in duplicate for each treatment. The suspensions were equilibrated at 50 °C for 1 h in a temperature-controlled water bath to allow complete hydration of the biomass. Following equilibration, the pH of each suspension was adjusted to approximately 8. Proteolytic treatments were then applied and carried out at 50 °C for 14 h under constant conditions. Seven experimental treatments were evaluated: (i) no protease addition, (ii) Alcalase^®^ added at 5% (*w*/*w*, relative to biomass protein content), (iii) Flavourzyme^®^ added at 5%, (iv) Formea^®^ Prime added at 5%, (v) Alcalase^®^ + Flavourzyme^®^ at 2.5% each, (vi) Alcalase^®^ + Formea^®^ Prime at 2.5% each, and (vii) Flavourzyme^®^ + Formea^®^ Prime at 2.5% each. Upon completion of the hydrolysis step, the reaction mixtures were passed through a 1 mm mesh sieve to separate liquid fractions from insoluble residues. The filtrates were retained at 4 °C for subsequent processing. To further recover solubilized compounds, the remaining solid fractions were subjected to alkaline extraction. This step was performed in three consecutive cycles using 80 mL of an alkaline reducing solution containing sodium hydroxide (4 g L^−1^) and N-acetyl-L-cysteine (1 g·L^−1^). Each extraction was conducted at ambient temperature with orbital agitation (130 rpm) for 1.5 h. After each cycle, solids were collected and re-extracted using freshly prepared alkaline solution. The supernatants from all alkaline cycles were combined with their corresponding enzymatic hydrolysates. The pooled treatments were adjusted to a final pH between 8.5 and 9.0 to enhance the solubility of proteins, peptides, and free amino acids. Samples were then frozen (−20 °C for 2 h followed by −80 °C for 24 h) prior to lyophilization using a freeze dryer (LaboGene A/S, Allerød, Denmark). The dried treatments were sealed in plastic bags and stored at −80 °C until further analyses.

### 4.4. Screening Crude Treatments and Fractions via α-Glucosidase Inhibition Assay

The α-glucosidase inhibitory activity of crude treatments and fractions was evaluated using a colorimetric microplate-based assay. The α-glucosidase from *S. cerevisiae* was used as a preliminary screening model, as it is widely employed in inhibitor evaluation studies. Briefly, 40 μL of each treatment solution was transferred into individual wells of a 96-well microplate. Acarbose was included as a reference inhibitor, while sodium phosphate buffer (0.1 M, pH 6.8) was used as blank. The enzymatic reaction was initiated by adding 40 μL of α-glucosidase solution (0.5 U·mL^−1^) to each well, followed by gentle mixing and incubation at 25 °C for 3 min. Subsequently, 20 μL of *p*-nitrophenyl-α-D-glucopyranoside substrate solution (0.5 U·mL^−1^) was introduced, and the plate was mixed again to ensure uniform reaction conditions. After allowing the reaction to proceed, enzymatic activity was terminated by the addition of 100 μL of sodium carbonate solution (0.1 M) to each well. The absorbance was measured at 405 nm using an Eon^TM^ microplate reader (BioTek Instruments, Inc., Winooski, VT, USA) following a 10 min incubation period. The percentage inhibition of α-glucosidase was calculated as follows:

$$\alpha -glucosidase\;inhibition\;(\%)=\left(1-\frac{Absorbance\;of\;sample}{Absorbance\;of\;blank}\right)\times 100$$

The α-glucosidase inhibitory activity of crude treatments and fractions was assessed at four concentrations (0.5, 1, 2, and 4 mg·mL^−1^). Sample concentrations needed to achieve 50% α-glucosidase inhibition (IC_50_ values) were determined by drawing dose–response curves.

### 4.5. Stepwise Membrane Filtration of Selected Treatment

Molecular weight-based separation of the selected crude treatment based on α-glucosidase inhibition results was performed using a stirred ultrafiltration system with a working volume of 300 mL (Millipore, Jaffrey, NH, USA). Regenerated cellulose Ultracel^®^ membranes (76 mm diameter; Millipore, Jaffrey, NH, USA) with nominal cut-off values of 5, 3, and 1 kDa were employed to obtain peptide fractions of different size ranges. The fractionation process was conducted sequentially, beginning with filtration through the 5 kDa membrane under pressurized nitrogen (5 bar). The permeate generated from this step was subsequently processed through membranes with decreasing molecular weight cut-offs (3 kDa followed by 1 kDa) to achieve further size-based separation of the soluble components. This stepwise ultrafiltration strategy resulted in four molecular weight fractions corresponding to >5 kDa, 3–5 kDa, 1–3 kDa, and <1 kDa. All collected fractions were concentrated by freeze-drying and stored at −80 °C under sealed conditions until further biochemical and in silico analyses.

### 4.6. Purification of Peptides via Fast Protein Liquid Chromatography (FPLC)

Following ultrafiltration, peptide-enriched fractions were further refined using size exclusion chromatography performed on an ÄKTA Pure FPLC system. Separation was carried out on a HiPrep 16/60 Sephacryl S-500 HR column (GE Healthcare, Chicago, IL, USA) with phosphate-buffered saline (PBS) as the mobile phase at 1 mL min^−1^. Prior to injection, samples were clarified by centrifugation, and 1 mL of supernatant was applied to the column. The elution profile was monitored, and the major peak corresponding to peptide-containing material was collected, thereby removing higher molecular weight impurities such as intact proteins or aggregates. This purification step yielded a more homogeneous peptide preparation for downstream identification and computational analyses. It should be noted that the enzyme inhibition screening assays were conducted using membrane-filtrated fractions, whereas the FPLC-purified peptides were reserved for subsequent characterization.

### 4.7. Identification of Peptides Through Liquid Chromatography-Tandem Mass Spectrometry (LC-MS/MS)

Peptide fractions were acidified to 1% trifluoroacetic acid (TFA), desalted using SOLAµ SPE plates (Thermo Fisher Scientific, Waltham, MA, USA), and dried in a SpeedVac. The dried peptides were reconstituted in 2% acetonitrile/1% TFA and quantified by absorbance (Nanodrop, Thermo Fisher Scientific). Samples were then separated by reversed-phase liquid chromatography using a Thermo EasyLC 1200 system fitted with a C18 trap column and an EasySpray analytical column (Thermo Fisher Scientific). Peptides were eluted with a 70 min gradient from 6% to 60% acetonitrile in 0.1% formic acid at a flow rate of 250 nL min^−1^ and analyzed on a Q-Exactive mass spectrometer (Thermo Fisher Scientific) operated in data-dependent acquisition (Top10) mode. Raw data were processed using Proteome Discoverer 2.4 (Thermo Fisher Scientific). Database searches were performed with SequestHT against the UniProt reference proteome supplemented with relevant recombinant sequences. Dynamic modifications included oxidation (M), acetylation (K), N-terminal acetylation, and methionine loss. Peptide-spectrum matches were filtered using a delta Cn cutoff of 0.05.

### 4.8. Computational Prediction of Peptide Bioactivity, Physicochemical Properties, and Safety

Peptide sequences identified by LC-MS/MS were evaluated using computational prediction tools to assess their potential bioactivity and safety profile. Bioactivity probability was estimated with PeptideRanker (http://distilldeep.ucd.ie/PeptideRanker/) (accessed on 27 June 2025), and sequences scoring above 0.6 were advanced for further analysis. Toxicity and allergenicity were predicted using ToxinPred (https://webs.iiitd.edu.in/raghava/toxinpred/protein.php), AllerCatPro 2.0 (https://allercatpro.bii.a-star.edu.sg/), and AllergyPred (https://allergypred.charite.de/AllergyPred/) (all accessed on 28 June 2025). Only peptides classified as non-toxic and non-allergenic by all tools were retained. Potential antidiabetic activities were examined using BIOPEP-UWM (https://biochemia.uwm.edu.pl/en/biopep-uwm-2/) (accessed on 14 July 2025), focusing on known motifs associated with α-glucosidase inhibition. Top candidates from this computational screening were selected for subsequent molecular docking studies. Physicochemical characterization of selected α-glucosidase-inhibiting peptides was performed using a formular Calculate Pi of a Peptide (https://sourcetable.com/calculate/how-to-calculate-pi-of-a-peptide) (accessed on 12 February 2026).

### 4.9. Molecular Docking Simulations

To explore the binding potential of the selected three peptides with predicted antidiabetic activity toward α-glucosidase, molecular docking simulations were carried out using the web-based platforms ClusPro 2.0 (https://cluspro.org/) (accessed on 10 January 2026) and Swiss-Model (https://swissmodel.expasy.org/) (accessed on 15 December 2025). Three-dimensional peptide conformations were predicted using PEP-FOLD4 (https://bioserv.rpbs.univ-paris-diderot.fr/index.html) (accessed on 12 December 2025) and converted into suitable docking formats. The sequence of human α-glucosidase was obtained from Protein Data Bank (https://www.rcsb.org/; PDB ID: 5NN3) (accessed on 12 December 2025). Peptides were docked individually to the enzyme, and the generated binding poses were evaluated based on predicted binding affinity, stability, and proximity to the active site. The most promising candidates, defined as those exhibiting the lowest predicted binding energies and favorable binding orientations, were selected for further discussion. Docking complexes were visualized using Discovery Studio Visualizer (BIOVIA, Dassault Systèmes, San Diego, CA, USA).

### 4.10. Statistical Analyses

Data were examined using one-way analysis of variance (ANOVA), and mean differences among groups were determined using the Bonferroni test. For comparisons between two groups, Welch’s unpaired two-tailed *t*-test was applied. Statistical analyses were performed with OriginPro 2023 (OriginLab Co., Northampton, MA, USA), and *p* < 0.05 was considered statistically significant.

## 5. Conclusions

This study demonstrates that sequential enzymatic/alkaline treatment of algal biomass, particularly using Alcalase^®^, generates low-molecular-weight peptides with potent α-glucosidase inhibitory activity. Membrane fractionation enriched bioactive components, with the <1 kDa fraction exhibiting the strongest inhibition. Three peptides RADIPFRRA, DGIAEAWLG, and FWSQIFGVAF displayed distinct binding modes in silico, with RADIPFRRA and DGIAEAWLG acting via competitive inhibition at the catalytic cleft, while FWSQIFGVAF likely exerts allosteric modulation through peripheral binding. Physicochemical differences among these peptides, including charge, isoelectric point, and hydrophobicity, were consistent with their observed docking behavior. Overall, these findings highlight the structural and functional diversity of algal-derived peptides and their potential as natural modulators of carbohydrate metabolism. The coexistence of competitive and allosteric inhibitory mechanisms offers multiple avenues for designing peptide-based therapeutics or functional food ingredients targeting α-glucosidase. Future work should focus on in vitro and in vivo validation to assess efficacy, bioavailability, and safety of these peptides in metabolic regulation.

## Figures and Tables

**Table 1:** α-Glucosidase inhibition (%) and IC_50_ values of enzymatic/alkaline red seaweed treatments produced using individual proteases and combinations thereof.

	4 mg·mL^−1^	2 mg·mL^−1^	1 mg·mL^−1^	0.5 mg·mL^−1^	IC_50_ (mg·mL^−1^)
No Protease	31.04 ± 0.48 ^d,w^	18.98 ± 1.11 ^c,x^	13.97 ± 1.21 ^e,y^	4.65 ± 0.67 ^d,z^	-
Alcalase^®^	68.19 ± 1.01 ^a,w^	45.43 ± 4.60 ^a,x^	30.74 ± 1.62 ^a,y^	25.94 ± 1.37 ^a,y^	2.48 ^a^
Flavourzyme^®^	41.24 ± 1.28 ^c,w^	31.65 ± 1.70 ^b,x^	22.94 ± 0.23 ^b,y^	17.32 ± 1.12 ^b,z^	-
Formea^®^ Prime	33.30 ± 1.81 ^d,w^	21.91 ± 0.82 ^c,x^	15.10 ± 0.92 ^de,y^	10.26 ± 0.65 ^c,z^	-
Alcalase^®^ + Flavourzyme^®^	54.57 ± 0.79 ^b,w^	41.49 ± 1.32 ^a,x^	20.08 ± 1.11 ^bc,y^	13.31 ± 1.31 ^bc,z^	3.36 ^b^
Alcalase^®^ + Formea^®^ Prime	52.97 ± 1.54 ^b,w^	41.70 ± 0.92 ^a,x^	17.58 ± 0.18 ^cd,y^	10.70 ± 1.65 ^c,z^	3.46 ^b^
Flavourzyme^®^ + Formea^®^ Prime	39.82 ± 2.29 ^c,w^	21.24 ± 1.09 ^c,x^	14.08 ± 1.40 ^e,y^	9.41 ± 3.65 ^cd,y^	-

Data were expressed as mean ± standard deviation (*n* = 3). The superscripts a–e indicate significant differences among the treatments at each concentration tested or among the IC_50_ values, and the superscripts w–z indicate significant differences among the concentrations for individual treatments (*p* < 0.05). IC_50_ values were calculated when inhibition reached 50% within the tested concentration range (up to 4 mg·mL^−1^).

**Table 2:** α-Glucosidase inhibition (%) and IC_50_ values of different molecular weight fractions of Alcalase^®^-assisted treatment.

	4 mg·mL^−1^	2 mg·mL^−1^	1 mg·mL^−1^	0.5 mg·mL^−1^	IC_50_ (mg·mL^−1^)
>5 kDa	24.16 ± 1.10 ^c,w^	16.73 ± 0.82 ^c,x^	7.49 ± 1.04 ^c,y^	1.04 ± 0.18 ^c,z^	-
3–5 kDa	26.83 ± 0.92 ^c,w^	17.53 ± 1.51 ^c,x^	9.20 ± 1.79 ^c,y^	2.95 ± 0.42 ^c,z^	-
1–3 kDa	43.56 ± 0.29 ^b,w^	30.07 ± 1.00 ^b,x^	20.49 ± 1.52 ^b,y^	11.57 ± 1.82 ^b,z^	-
<1 kDa	70.10 ± 1.18 ^a,w^	53.46 ± 2.68 ^a,x^	38.56 ± 2.05 ^a,y^	25.04 ± 1.43 ^a,z^	1.94

Data were expressed as mean ± standard deviation (*n* = 3). The superscripts a–c indicate significant differences among the treatments at each concentration tested, and the superscripts w–z indicate significant differences among the concentrations for individual treatments (*p* < 0.05). IC_50_ values were calculated when inhibition reached 50% within the tested concentration range (up to 4 mg·mL^−1^).

**Table 3:** Predicted physicochemical properties of peptides identified from Alcalase^®^/alkaline treatments of *P. palmata*.

Sequence	Number of Residues	Molecular Weight (g·mol^−1^)	Isoelectric Point	Net Charge at pH 7
FWSQIFGVAF	10	1201.39	5.52	−0.24
RADIPFRRA	9	1101.26	11.70	+1.76
DGIAEAWLG	9	931	3.67	−2.24

## Data Availability

The data acquired in this study can be obtained at request.

## Supplementary Materials

The following supporting information can be downloaded at: https://www.mdpi.com/article/10.3390/md24030091/s1. Table S1. Predicted bioactive peptides derived from *Palmaria palmata*, their bioactivity scores, and corresponding parent protein accession numbers. Peptides selected for α-glucosidase inhibitory activity are highlighted.
