# Supplementary material for: Macroalgal Peptides with Predicted α-Glucosidase Inhibitory Activity: Preparation and Molecular Docking
Source: Mar Drugs. 2026 Feb 26;24(3):91. doi: 10.3390/md24030091 (PMC13027621; doi:10.3390/md24030091)
Supplement: Supplementary file 1 [file marinedrugs-24-00091-s001.zip › marinedrugs-4182931-supplementary.pdf]

Table S1. Predicted bioactive peptides derived from *Palmaria palmata*, their bioactivity scores, and corresponding parent protein accession numbers. Peptides selected for α-glucosidase inhibitory activity are highlighted.

Sequence	Peptide Ranker Score	Protein Accession
KGAMPAFGGRL	0.930436	A0A1C9CHB8
YAMPPYAFM	0.916668	A0A1C9CGW2
SASLWERFC	0.901258	O98733
FWSQIFGVAF	0.89401	A0A1C9CHE2
FFSGYRPQF	0.891886	A0A1C9CHG7
MATKFPKF	0.81546	A0A1C9CGW2
RPASEPGFGLL	0.815445	A0A1C9CH28
SEPGFGLLR	0.813794	A0A1C9CH28
QAVYMKFPFT	0.808071	A0A1C9CH48
ERFLSQPFF	0.801814	A0A1C9CH09
WAPGGGDVRFI	0.769119	A0A455TML9
NRPASEPGFGL	0.758887	A0A1C9CH28
CARDIGYYL	0.758123	A0A1C9CH48
CYRDVDHYMRL	0.757031	A0A1C9CH34
GAAQAVYMKF	0.755674	A0A1C9CH48
PEACAAILW	0.753151	A0A455TN39
ADFAQQLGSVICM	0.748183	A0A455TN66
KGFLFARNSRL	0.744664	A0A1C9CGZ6
FSDQYDRFK	0.740842	A0A1C9CGZ8
FFEQDWASLR	0.740131	A0A1C9CH59
PEALFKPEMLG	0.737964	Q8GU34
GLVGPDAGKEMGL	0.716686	M1VJV1
ESFNIPAFY	0.708849	Q8GU34
RADIPFRRA	0.707052	A0A1C9CHG0
SWDGPALVVFT	0.706342	A0A1C9CH79
QIFLSGDLF	0.7058	A0A1C9CH13
ALAEYFMYKGK	0.703625	A0A1C9CH13
YRLGMRPWI	0.678017	O98733
AQIPVGDAFLGRV	0.67481	A0A1C9CH13
DVNLPQGIFF	0.674479	A0A5Q3RCF2
QLQPIFAQW	0.669789	A0A1C9CGZ6
DGIAEAWLG	0.666104	A0A1C9CHG0
GGQQLFQKR	0.665128	M1UZ22
KAADKIFTGG	0.663255	A0A1C9CGY2
AIDSMIPIGRG	0.656497	A0A1C9CH13
SGDLFNSGIRPAI	0.643587	A0A1C9CH13
MGYWDPEHVIL	0.642326	A0A1C9CH59
CLRDLDYYL	0.638864	A0A1C9CH06
LVGPDAGKEMGL	0.636844	M1VJV1
VSLFLGFHTL	0.634707	A0A1C9CGW2
CIRDLDYYL	0.633154	M1VJV1
RIDLAGRDLTGWM	0.630624	Q8GU34

GGVSVFGGVG	0.623333	A0A1C9CH09
SGSPGLHMS	0.6218	A0A1C9CH62
QEQAPKAGDPALF	0.616025	A0A1C9CH69
FSENIVPYRR	0.61216	A0A1C9CGY2
YRDVDHYMRL	0.611581	A0A1C9CH34
LDLWKDITF	0.607617	A0A5Q3RCF2
ADWQPGDRT	0.605477	A0A1C9CGZ8
WGGGKIYY	0.603039	A0A1C9CH86
DAGKEMGLYF	0.601144	M1VJV1
